# Abdominal obesity and other risk factors largely explain the high CRP in Indigenous Australians relative to the general population, but not gender differences: a cross-sectional study

**DOI:** 10.1186/1471-2458-10-700

**Published:** 2010-11-15

**Authors:** Allison M Hodge, Louise Maple-Brown, Joan Cunningham, Jacqueline Boyle, Terry Dunbar, Tarun Weeramanthri, Jonathan Shaw, Kerin O'Dea

**Affiliations:** 1 University of Melbourne, Department of Medicine, St Vincent's Hospital, Melbourne, Australia; 2 Menzies School of Health Research, Institute of Advanced Studies, Charles Darwin University, Darwin, Australia; 3 Division of Medicine, Royal Darwin Hospital, Darwin, Australia; 4 Faculty of Education, Health and Science and Graduate School of Health Practices, Charles Darwin University, Australia; 5 Public Health Division, WA Health, Perth, Australia; 6 Baker IDI Heart and Diabetes Institute, Melbourne, Australia; 7 Sansom Institute for Health Research, University of South Australia, Australia

## Abstract

**Background:**

Previous studies reported high C-reactive protein (CRP) levels in Indigenous Australians, which may contribute to their high risk of cardiovascular disease. We compared CRP levels in Indigenous Australians and the general population, accounting for obesity and other risk factors.

**Methods:**

Cross-sectional study of CRP and risk factors (weight, height, waist and hip circumferences, blood pressure, lipids, blood glucose, and smoking status) in population-based samples from the Diabetes and Related conditions in Urban Indigenous people in the Darwin region (DRUID) study, and the Australian Diabetes, Obesity and Lifestyle study (AusDiab) follow-up.

**Results:**

CRP concentrations were higher in women than men and in DRUID than AusDiab. After multivariate adjustment, including waist circumference, the odds of high CRP (>3.0 mg/L) in DRUID relative to AusDiab were no longer statistically significant, but elevated CRP was still more likely in women than men. After adjusting for BMI (instead of waist circumference) the odds for elevated CRP in DRUID participants were still higher relative to AusDiab participants among women, but not men. Lower HDL cholesterol, impaired glucose tolerance (IGT), and higher diastolic blood pressure were associated with having a high CRP in both men and women, while current smoking was associated with high CRP in men but not women.

**Conclusions:**

High concentrations of CRP in Indigenous participants were largely explained by other risk factors, in particular abdominal obesity. Irrespective of its independence as a risk factor, or its aetiological association with coronary heart disease (CHD), the high CRP levels in urban Indigenous women are likely to reflect increased vascular and metabolic risk. The significance of elevated CRP in Indigenous Australians should be investigated in future longitudinal studies.

## Background

C-reactive protein (CRP) is an acute phase protein synthesised predominantly by the liver [1]. Epidemiological evidence suggests that CRP is associated with coronary heart disease (CHD) [2]; diabetes and the metabolic syndrome [3]. High CRP has been reported in Indigenous Australians, and may contribute to or be a marker for their elevated risk of CHD [4]. In a remote Indigenous population, over 50% of participants had a CRP above 3.0 mg/L, representing high risk [4], compared with only 25% in the Busselton Study (mainly non-Indigenous) [5]. Approximately 60% of high CRP in Busselton could be attributed to smoking, body mass index (BMI), blood pressure, diabetes, total cholesterol, triglycerides and low HDL cholesterol [5]. These tend to be worse in Indigenous Australians and could contribute to elevated CRP concentrations [6,7].

Other studies have variously shown that women had CRP concentrations higher than men [8-11] (including a study of Indigenous Australians [4]), similar to men [5] or lower than men [12]. Oral contraceptive and hormone replacement therapy use are both associated with higher CRP [5,8,9,13] and may contribute to higher levels sometimes seen in women. Traditional risk factors, particularly obesity, are also associated with CRP [4,5,8-13]. Socioeconomic factors have also been related to levels of CRP [14] and other inflammatory markers [15,16].

The aim of this study was to compare CRP in an urban Indigenous population and the general Australian population, and to determine the contribution of other risk factors to any differences observed. In view of the different associations between BMI and CRP in men and women previously observed in Aboriginal Australians [4], men and women were analysed separately.

## Methods

### Study population

The Diabetes and Related conditions in Urban Indigenous people in the Darwin region (DRUID) study was established to provide information on the prevalence of diabetes and diabetes-related complications among Indigenous Australians in an urban area [17]. Eligible participants were aged 15 years or more who identified as Aboriginal and/or Torres Strait Islander, had lived within a defined geographic region in and around the city of Darwin for at least 6 months, and did not live in an institutional dwelling. A sampling frame was not available, but using available data we estimate that 14% of eligible persons participated. Comparison with national census data and data from the Northern Territory Department of Health and Community Services suggested that the participants were more likely to be female and participating females tended to be older than the target population [17]. A total of 1004 people had at least one measurement performed during 2003-05 [17].

The Australian Diabetes, Obesity and Lifestyle study (AusDiab) is a national, population-based, longitudinal survey of diabetes and associated risk factors in Australians ≥25 years of age, which commenced in 1999. Of the original cohort (n = 11,247, response rate 37%, 10,788 participants were eligible (ie not deceased, withdrawn from study, moved overseas, moved to high-care nursing facility or with chronic/terminal disease) for the 5-year follow-up in 2004-2005, when CRP was measured. A total of 6537 (60.6%) AusDiab participants returned for follow-up assessment. Compared with those who did not attend follow-up, attendees were likely to be better educated, healthier and not to smoke [18].

The current analysis was limited to people aged 30-64 years, as there were no AusDiab participants aged <30 and very few DRUID participants aged ≥65. Those with incomplete data were excluded from analysis, as were women who were pregnant (n = 13), using hormone replacement therapy (n = 311) or the oral contraceptive pill (n = 184) as these women might be expected to have elevated CRP levels [5]. The remaining 512 DRUID participants and 2823 AusDiab participants were included.

### Study design

AusDiab and DRUID followed similar protocols. Eligible participants who gave consent underwent a health examination including the collection of blood and urine samples, clinical and anthropometric measurements, and administration of questionnaires.

A 2-hour oral glucose tolerance test was administered to all participants who gave consent, with the exception of those taking tablets or insulin for previously diagnosed diabetes, and those who were pregnant. Participants were classified as having diabetes if they met any of the following criteria: 1) fasting plasma glucose (FPG) ≥7.0 mmol/L; 2) 2-hour plasma glucose (2 hPG) ≥11.1 mmol/L; or 3) previously diagnosed as having diabetes and currently taking tablets and/or insulin for diabetes. Among participants who were not currently taking tablets and/or insulin for diabetes, impaired glucose tolerance (IGT) was considered present if FPG <7.0 mmol/L and 2 hPG ≥ 7.8 and <11.1 mmol/L; impaired fasting glucose (IFG) was considered present if FPG was ≥ 6.1 and <7.0 mmol/L, and 2 hPG was less than 7.8 mmol/L [19]. Fifty two participants who were not originally classified but had fasting glucose < 6.1 mmol/l were considered to have normal glucose tolerance.

Plasma high sensitivity (hs)-CRP measurement in DRUID was performed at the Clinical Trials Laboratory (Flinders Medical Centre, Bedford Park, SA) by immunoturbidimetry using a Hitachi 917 analyzer (Hitachi Ltd., Tokyo, Japan) and Roche reagents. CRP in AusDiab was measured by chemiluminescent enzyme immunoassay (Immulite DPC 2000, CA). Details of data collection including blood sampling, blood pressure measurement, anthropometry and biochemical methods are described in detail elsewhere [17,20].

### Statistical analysis

Where CRP was treated as a continuous variable it was log_n _transformed. A categorical variable was also created dividing CRP according to the American Heart Association (AHA) recommendations [21]: low risk (<1.0 mg/L); average risk (≥1.0 to 3.0 mg/L); and high risk (≥3.0 mg/L), which was further divided into ≥3.0 & < 10.0 and ≥10.0 mg/L. The AHA recommends that CRP levels above 10 mg/L are likely due to acute inflammation and should be ignored [21]. However, in a remote Indigenous group, CRP levels across the range were maintained over a median 829 days [22], and we have not excluded these values.

Associations between geometric mean CRP levels and likely confounders (age and obesity) were plotted for the 4 groups: AusDiab men, AusDiab women, DRUID men and DRUID women. BMI was categorised as: underweight (BMI <18.5 kg/m^2^); normal weight (BMI 18.5-24.9 kg/m^2^); overweight (BMI 25-29.9 kg/m^2^); and obese (BMI 30+ kg/m^2^) [23], but because of the small number of people in the underweight category, the first two groups were collapsed. Waist circumference was categorised as: low risk for obesity associated metabolic complications (<94 cm for men & < 80 cm for women); increased risk (94-101.9 cm for men & 80-87.9 cm for women); and substantially increased risk (> 102 cm for men & > 88 cm for women) [23].

Several previous studies have reported different associations between CRP and obesity or other risk factors for men and women [4,9-11]; hence all analyses are performed separately for men and women. Interaction terms were calculated for measures of obesity (waist and BMI) by gender and tested in multivariate models to determine whether in fact gender modified the associations between obesity and CRP. Logistic regression with CRP >3.0 (yes/no) as the dependent variable was used to identify variables associated with elevated CRP. Variables included in these models were: population (AusDiab, DRUID), age group (30-34, 35-44, 45-54, 55-64), total cholesterol (mmol/L), log_n _triglycerides (mmol/l), HDL cholesterol (mmol/L), systolic blood pressure (mmHg), diastolic blood pressure (mmHg), glucose tolerance status (normal, IFG, IGT, diabetes), smoking status (smoker, non-smoker) and either BMI (kg/m^2^) or waist circumference (cm) as continuous variables. Triglyceride values were natural log transformed to improve normality. Variables other than triglycerides were normally distributed. Models were also computed stratifying on population, to evaluate the association of gender with elevated CRP. Models were repeated with CRP > 10 mg/L (yes/no) as the outcome.

### Ethical approval

Ethics approval for DRUID was given by the combined Human Research Ethics Committee of Northern Territory Department of Health and Community Services and Menzies School of Health Research, Darwin. The ethics committee of the International Diabetes Institute approved the AusDiab study. Informed consent was obtained from all participants.

## Results

DRUID participants were on average 6-8 years younger than AusDiab participants (Table 1). Mean BMIs were all in the overweight range; DRUID women had a mean around 2 units higher than AusDiab women. The differences in waist to hip ratio (WHR) and waist circumference between the two groups of women were marked. DRUID participants were more likely to have diabetes and more likely to smoke than AusDiab participants. DRUID men and women had mean CRP values in the high-risk range ≥ 3.0 mg/L, and DRUID women had a mean around twice that of AusDiab women.

**Table 1:** Characteristics of study populations.

	DRUID		AUSDIAB	
**Variable**	**Men n = 167**	Women* n = 345	**Men n = 1391**	Women* n = 1432

*Mean ± SD*				
Age (yrs)	43.2 ± 8.7	44.5 ± 8.9	50.9 ± 8.5	50.3 ± 8.4
BMI (kg/m^2^)	28.8 ± 5.6	29.7 ± 7.7	28.0 ± 4.5	27.7 ± 6.0
WHR	0.98 ± 0.07	0.90 ± 0.10	0.94 ± 0.07	0.82 ± 0.06
Waist (cm)	99.9 ± 14.1	97.5 ± 17.1	98.2 ± 12.0	87.2 ± 13.8
Systolic blood pressure (mmHg)	124.5 ± 16.4	117.8 ± 16.8	124.1 ± 17.7	115.4 ± 17.7
Diastolic blood pressure (mmHg)	79.5 ± 10.3	75.5 ± 9.3	73.3 ± 9.1	66.0 ± 9.7
Total cholesterol (mmol/L)	5.40 ± 1.33	5.17 ± 0.94	5.19 ± 0.94	5.25 ± 0.97
HDL (mmol/L)	1.02 ± 0.32	1.17 ± 0.33	1.25 ± 0.31	1.56 ± 0.39
*Geometric mean(95%CI)*				
Fasting glucose (mmol/L)	6.04(5.77-6.32)	5.85(5.67-6.04)	5.48(5.44-5.52)	5.23(5.19-5.27)
CRP (mg/L)	3.43(2.68-3.63)	4.65(4.10-5.27)	1.94(1.84-2.04)	2.16(2.03-2.29)
Triglycerides (mmol/L)	2.13(1.93-2.35)	1.58(1.49-1.67)	1.43(1.39-1.47)	1.10(1.07-1.13)
Fibrinogen	3.69(3.51-3.89)	3.82(3.68-3.97)	2.70(2.67-2.74)	2.86(2.82-2.89)
Fasting Insulin (mU/L)**	9.11(8.16-10.18)	10.41(9.54-11.37)	7.56(7.31-7.83)	6.94(6.72-7.18)
HOMA-IR**	2.45(2.15-2.78)	2.71(2.45-3.00)	1.84(1.77-1.91)	1.61(1.55-1.67)
*Percentages*				
Glucose tolerance				
Diabetes	23.3	25.2	6.8	5.4
IFG/IGT	19.8	16.5	12.8	9.5
Normal	56.9	58.3	80.4	85.1
Smoking status				
Current	45.5	42.9	13.0	8.9
Former	28.1	22.0	33.9	27.6
Never	21.0	27.2	49.4	59.6

*Women who are pregnant, using HRT or oral contraceptives are excluded.

**People with known diabetes on insulin (AusDiab), or insulin/tablets (DRUID) are excluded from these estimates.

**Figure 1:**
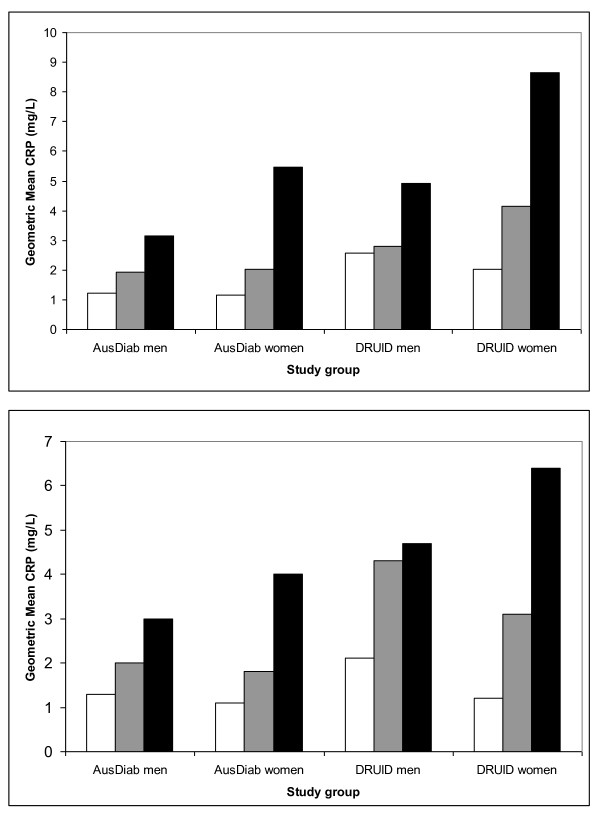
**Geometric mean CRP**. a) Geometric mean CRP by BMI category and study group. <25 kg/m^2 ^white bars. 25-30 kg/m^2 ^grey bars. 30+ kg/m^2 ^black bars. b) Geometric mean CRP by waist circumference category and study group. <94 cm men; <80 cm women white bars. 94-101.9 cm men; 80-87.9 cm women grey bars. >102 cm men; 88 cm women black bars

Figure 1 shows mean CRP levels according to categories of BMI (a) and waist circumference (b) for each study group. Among those in the smallest category of waist circumference, DRUID women show little evidence of elevated CRP. With increasing obesity, mean CRP levels increased more steeply for DRUID women than for other groups. Excluding the 8 men and 29 women with BMI less than 18.5 kg/m^2 ^did not appreciably change the results.

**Figure 2:**
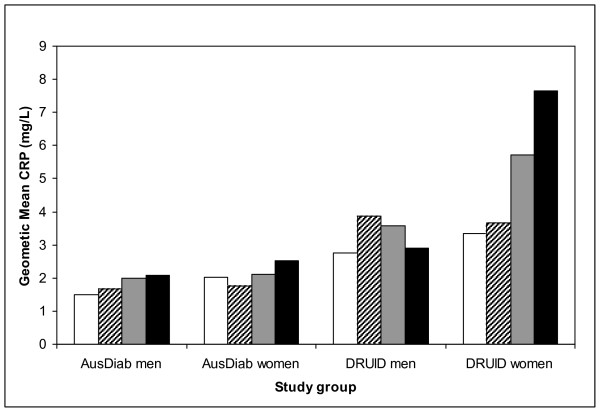
**Geometric mean CRP by age and study group**. 30-34 years white bars. 35-44 years diagonal striped bars. 45-54 years grey bars. 55-64 years black bars

Figure 2 shows mean CRP levels according to age for each group. In general, CRP increased slightly with age up to 65 years, most notably in DRUID women; but for DRUID men CRP was highest among those aged 35-44 years.

In unadjusted models, the OR for elevated CRP in DRUID men relative to AusDiab men was 2.58 (95%CI 1.84-3.57); for women the OR was 2.89 (2.26-3.70). Interactions between measures of obesity (BMI and waist) and gender were significant (p = 0.003 for both BMI and waist circumference) in multivariate models. Table 2 shows odds ratios (ORs) for multivariate logistic regression with elevated CRP (≥3.0 mg/L, yes/no) as the dependent variable, and waist circumference as the measure of obesity. After adjustment, the OR for elevated CRP in DRUID relative to AusDiab was no longer significantly different to unity. For both men and women, waist circumference, HDL cholesterol (inverse), diastolic blood pressure, and glucose tolerance were associated with elevated CRP. Smoking in men, but not women was also associated with elevated CRP. Using forward stepwise logistic regression revealed that obesity, whether measured as waist circumference or BMI, was the first variable to be entered in models for men and women and for DRUID and AusDiab participants.

**Table 2:** Odds ratios and 95% confidence intervals (OR, 95% CI) in multivariate models for factors associated with the presence of elevated CRP (>3.0 mg/L) in DRUID and AusDiab.

	Men		Women	
**Variable**	**OR**	**95%CI**	**OR**	**95%CI**

**Study**				

AusDiab	1.00		1.00	

DRUID	1.30	0.86-1.98	1.04	0.72-1.50
**Age**				
30-34	1.00		1.00	
35-44	0.77	0.45-1.31	0.85	0.51-1.43
35-54	0.73	0.43-1.22	0.86	0.52-1.43
55-64	0.78	0.46-1.32	0.91	0.54-1.56
Waist circumference (cm)	1.06	1.05-1.07	1.07	1.06-1.09
**Glucose tolerance**				
Normal	1.00		1.00	
IFG	1.47	0.89-2.42	1.61	0.86-3.03
IGT	1.70	1.14-2.53	2.60	1.62-4.16
Diabetes	2.11	1.38-3.24	1.43	0.90-2.25
**Smoking status**				
Current	1.00		1.00	
Non-Smoker	0.47	0.34-0.64	1.00	0.71-1.41
HDL (mmol/L)	0.42	0.25-0.69	0.61	0.41-0.91
Total cholesterol (mmol/L)	1.08	0.94-1.23	1.07	0.93-1.22
Log_n _triglycerides (mmol/l)	0.94	0.71-1.26	1.28	0.93-1.78
Diastolic BP (mmHg)	1.02	1.00-1.04	1.02	1.00-1.04
Systolic BP (mmHg)	0.99	0.98-1.00	1.00	0.99-1.01

Stratifying by population indicated that for both DRUID and AusDiab participants, women were more likely to have an elevated CRP than men, after multivariate adjustment: OR for women relative to men in AusDiab 4.31 (95%CI 3.47-5.35), and DRUID 2.95 (95%CI 1.82-4.78). If BMI were used as the measure of obesity in the multivariate model, population remained as a significant determinant of CRP in women (OR for DRUID relative to AusDiab 1.47, 95%CI 1.01-2.13), but not for men (OR 1.28, 95%CI 0.85-1.94). Models were also computed including HOMA insulin resistance, but although this was associated significantly with CRP, its inclusion did not change the results appreciably.

Models including the same covariates were also fitted with the dependent variable as CRP ≥ 10.0 mg/L (yes/no). The OR for extreme CRP for DRUID males relative to AusDiab males was 1.55 (95%CI 0.83-2.89), and for DRUID females relative to AusDiab females 1.74 (95%CI 1.12-2.71). Stratifying by population, the OR for extreme CRP in women relative to men was 4.57 (95%CI 3.19-6.56) in AusDiab, and 3.62 (95%CI 2.07-6.35) in DRUID.

## Discussion

CRP concentrations in urban Indigenous participants from DRUID were higher than in the general population sample; Indigenous women had particularly elevated CRP levels. In men and women from both studies, CRP was strongly associated with waist circumference and BMI. Among those in the smallest waist circumference category, CRP levels were not elevated in DRUID women. After adjusting for waist circumference and other potential confounders, the risk of elevated CRP in DRUID was not significantly different to that for AusDiab, although women still had more elevated CRP than men. In men and women, having lower HDL cholesterol, impaired glucose tolerance (IGT), and higher diastolic blood pressure were also associated with having a high CRP, while current smoking was associated with high CRP in men but not women. The results for a CRP of 10.0 mg/l or above suggested that such extreme values were more common in DRUID women than AusDiab women, and in women than men, even after accounting for other risk factors.

The high CRP concentrations in this urban Indigenous population are consistent with data for an isolated Aboriginal community, where over 50% of participants had an elevated CRP [4]. Data from a remote Indigenous Island community were also compared with published data from different countries, demonstrating the relatively high CRP concentrations in the Indigenous group, in which 39% of men and 18% of women had CRP > 10 mg/L [24].

Wang and Hoy [24] showed that from around age 10, CRP levels rose with age and were always higher in females than males. Our unadjusted data also suggest increasing CRP with age, but in the multivariate analysis this was no longer seen. It is possible that the association between CRP and age was due to the increase in abdominal obesity with age, and so was lost once waist circumference was accounted for.

Our results, and the results from Shemesh et al [4], suggest that in the absence of obesity, women do not have elevated CRP relative to men. Similar CRP levels were also reported among lean Chinese men and women (median CRP men 0.84; women 0.93 mg/L) [25]. In a population-based study in Japan, men had higher CRP levels than women, but participants were relatively lean and CRP concentrations were extremely low, with a median for men of 0.16 mg/L and for women 0.09 mg/L [12]. Men and women from the Dallas Heart Study in the lowest tertile of fat mass (by DEXA) also did not show differences in CRP concentrations [26].

Other studies have shown steeper increases in CRP in association with risk factors in women relative to men. In an Israeli study [10]and in the Framingham Offspring Study [11], men with no metabolic syndrome components had a slightly higher CRP than women, but as the number of components increased, CRP rose more steeply in women than men, and there were significant interactions between number of components and sex [10,11]. Similarly in the Dallas Heart Study, CRP rose more steeply across BMI groups in women than in men, so that at BMI > 30 kg/m^2^, CRP levels in women were about twice those in men [9]. Among Aboriginal Australians from an isolated rural community there was a strong linear association between CRP and BMI in women, but no association in men [4].

Women using oral contraceptives or hormone replacement therapy tend to have higher levels than other women [5]. The possibility exists that endogenous estrogen is also associated with CRP [10], hence menopausal status may contribute to differences in CRP between the older AusDiab and younger DRUID women, but we do not have data to examine this. However, the data presented by Wang and Hoy [24] on CRP levels with age do not indicate any change in the association at an age where most women would reach menopause.

The significance of higher CRP in women than men across two very different populations is not clear. CRP is known to be associated with CHD, and typically men are considered to be at higher risk than women [27]. A recent review suggested that global inflammation, as indicated by elevated CRP, may predict cardiovascular outcomes in women better than the traditional risk factors identified in men [27]. Onat et al on the other hand found that CRP predicted CHD similarly in Turkish men and women, but diabetes in women only [3]. In either case, the extremely high CRP concentrations seen in the DRUID women could presage extremely high rates of diabetes and CHD, although no prospective data is available to confirm this.

It is of interest that in our multivariate models current smoking was associated with elevated CRP in men only. In a Turkish population-based study, smoking in women was actually associated with a lower CRP concentration than in non-smokers, while in men smokers had higher CRP than non-smokers [3].

In a recent study of Native Alaskans, CRP was found to be associated with pathogen burden, measured as IgG, IgA and IgM antibodies to *C. pneumoniae *and IgG antibodies to other pathogens [28]. High CRP levels seen in Indigenous Australians, particularly those above 10 mg/L, may be in part attributable to pathogen burden. For example, the prevalence of *H. pylori *in two Indigenous populations in Western Australia was extremely high, 91% in a remote community and 60% in an urban community, which is still twice what would be expected in the non-Indigenous Perth population [29]. High rates of acute otitis media related to infections with *H. influenzae *and *S. pneumoniae *are common in Indigenous children [30]. McDonald et al [31] found that higher CRP concentrations in a group of remote Australian Aborigines were associated with IgG seropositivity to *H. pylori *and *C. pneumoniae *and higher IgG titre for cytomegalovirus.

Our study has several limitations. The data are cross-sectional, and we are unable to comment on the representativeness of volunteer cohorts with low to moderate response rates. There is evidence that people who attended the AusDiab follow-up were healthier than those who did not attend [18], which would suggest they may have lower CRP concentrations than the more representative original sample. This may explain to some extent their lower unadjusted CRP levels relative to DRUID participants. However, the multivariate analysis accounted for many health related variables, both risk markers (lipids, glucose tolerance, blood pressure, obesity) and behavioural factors (smoking), which appeared to largely explain the differences between DRUID and AusDiab.

We are unable to account for the use of statins, which can reduce CRP levels [32], in DRUID. However, we do have data for all but 23 men and 18 women on lipid lowering medication, which includes statins. Adding an indicator variable for lipid-lowering medication did not markedly change the ORs for DRUID relative to AusDiab from those presented in Table 2; although in women, such drug use was associated with a reduced risk of high CRP.

The assay methods used for hs-CRP in both studies were different and we do not have data to directly evaluate their comparability. Roberts et al [33] compared 9 hs-CRP methods, including the DPC and Roche methods, with the DadeBehring BN II assay. Although both showed good concordance with the reference method, the Roche assay tended to give slightly higher results and correctly classified around 75% of 388 blood donors into quartiles based on the Dade method, while the DPC assay correctly classified around 95% [33]. Nonetheless, criteria proposed to identify high risk CRP concentrations do not specify the method, and are widely applied. The difference between assays could potentially contribute to higher hs-CRP values in DRUID using the Roche assay, but is unlikely to explain the large differences in crude CRP concentrations observed.

The main strength of our study is that we were able to combine data, collected using similar methods, from population based studies of the general population and an Indigenous Australian population.

## Conclusion

We have reported high levels of CRP in Indigenous Australians in the DRUID study, associated with adverse risk factor profiles. After adjusting for risk factors, in particular abdominal obesity, glucose tolerance, HDL cholesterol, diastolic blood pressure and smoking status, CRP levels in DRUID were no longer significantly different to AusDiab, but women in both groups had higher CRP values than men. Whether CRP is an independent risk factor for coronary heart disease is debatable [32,34]. Irrespective of its independence as a risk factor, or its aetiological association with CHD, the particularly high circulating CRP levels seen in the urban Indigenous women in association with increased abdominal fat are likely to reflect increased vascular disease and metabolic risk, and point to the need for interventions to address this. The significance of elevated CRP in Indigenous Australians should be investigated in future longitudinal studies to determine its significance as a predictor of future diabetes and CHD, and if relevant, reducing CRP may be an important target of interventions to improve the health of Indigenous Australians.

## Abbreviations

CRP: C-reactive protein; AusDiab: Australian Diabetes, Obesity and Lifestyle; DRUID: Diabetes and Related conditions in Urban Indigenous people in the Darwin region; BMI: body mass index; CHD: coronary heart disease; FPG: fasting plasma glucose; 2 hPG: 2 hour plasma glucose; IFG: impaired fasting glucose; IGT: impaired glucose tolerance; AHA: American Heart Association; DEXA: dual emission

## Competing interests

The authors declare that they have no competing interests.

## Authors' contributions

AH analysis plan, data analysis, manuscript preparation; LM-B data acquisition, drafting and revising manuscript; JC original study design and data acquisition, drafting and revising manuscript; JB data acquisition, drafting and revising manuscript; TD original study design, drafting and revising manuscript; TW original study design, drafting and revising manuscript; JS original study design, drafting and revising manuscript; KO'D original study design, drafting and revising manuscript. All authors have approved the final manuscript.

## Pre-publication history

The pre-publication history for this paper can be accessed here:

http://www.biomedcentral.com/1471-2458/10/700/prepub
